# Roles and responsibilities in substance use prevention in the school setting: views among Finnish school personnel representatives

**DOI:** 10.1080/02813432.2021.1935516

**Published:** 2021-06-17

**Authors:** Johanna Nordmyr, Anna K. Forsman

**Affiliations:** Faculty of Education and Welfare Studies, Health Sciences, Åbo Akademi University, Vaasa, Finland

**Keywords:** Qualitative research, focus groups, primary prevention, substance use prevention, mental health promotion, school, Finland

## Abstract

**Objective:**

This study explores the views of Finnish school personnel representatives regarding substance use prevention responsibilities.

**Design:**

Twenty-two focus groups were conducted within the scope of a regional intervention study in 2019. Qualitative content analysis was performed.

**Setting:**

Focus group interviews were conducted in the school setting.

**Subjects:**

Focus group participants included representatives for educational personnel and student welfare personnel working in basic education, general upper secondary education or vocational education settings.

**Main outcome measures:**

Views and experiences concerning roles and responsibilities in primary prevention of substance use.

**Results:**

Findings highlight the need for intersectoral efforts and intra-school collaboration in primary prevention efforts, but also in mental health promotion – on which the informants placed great emphasis. The health promotion leadership in schools, structural guidelines and the school curriculum could both challenge and support school personnel in their roles. An increased need to focus on the early years of life and related responsibilities of the homes was emphasized, along with the need to place more emphasis on health education in the first years of basic education, and responsibilities related to early risk identification.

**Conclusion:**

The findings highlight a need to develop structures and role clarity among school personnel, which can advance further development of intra-school and inter-sectoral collaboration in primary substance use prevention and mental health promotion. In the Finnish context, the successful implementation of relevant legislation, which some school representatives view as unclear or contravening, could be further supported.

**Key points**
- Views regarding responsibilities in primary substance use prevention in the school setting have been less researched in the Nordic countries:
- The importance of inter-sectoral and intra-school collaboration is emphasized among school personnel representatives, including the role of the homes
- Primary prevention and mental health promotion responsibilities are viewed as less clear than secondary and tertiary prevention responsibilities
- Structural guidelines concerning e.g. confidentiality aspects and curriculum features can both support and challenge school representatives in their roles

## Introduction

In the Nordic countries and Europe at large, adolescents’ use of licit and illicit substances and related risk behaviours form a continuing challenge [1]. Strategies to prevent substance use among children and adolescents encompass various interventions and policies, ranging from national policy to school and/or family-level interventions [2], with varying results shown for intervention effectiveness [3–7]. In contrast to evaluating substance use prevention programs, this study focuses on Finnish school personnel representatives’ perceptions of roles and responsibilities in prevention work aimed at children and adolescents – which bears implications for further development of primary prevention efforts in the school setting.

While several studies focusing on the views on roles and responsibilities in relation to substance use prevention have been conducted in a non-European context [8–13], less research with this focus has been conducted in Europe and the Nordic countries. Considering the available European research, one Irish study [14] showed that teachers deemed parents as most suited to educate and prevent student alcohol and cannabis use, followed by the students’ general practitioner and other health and welfare representatives, while teachers (themselves and other teachers) were viewed as slightly less suited to this task. However, the teachers did indicate a positive attitude toward the role of the school and the teachers’ involvement in alcohol and cannabis prevention. Another study compared Dutch and Norwegian parents’ perceptions regarding parental measures and governmental responsibility in relation to the prevention of adolescent substance use [15]. Both the Dutch and Norwegian parents, irrespective of the differing policy in their countries, emphasized parents’ responsibilities for taking measures against adolescents’ substance use.

No similar studies focusing on views on responsibilities in substance use prevention in the Finnish school setting have been published. However, one study examined the related theme of how parents and teachers in the Finnish and Russian Karelia region perceived home and school responsibilities associated with providing 10–11-year old children with information on different health topics. The topic of substance use was indicated by both parents and teachers to be a joint responsibility, albeit parents were given a larger role [16].

The present study was conducted in the Finnish context, where the Nordic welfare model is reflected in extensive services aimed at families and children. For school-aged children and adolescents, the Nordic and Finnish school setting constitutes an environment intended to support wellbeing and health [17]. In Finland, this includes the formalization of health education as a stand-alone subject (as opposed to being integrated with, e.g. physical education) in the National Core Curriculum for basic education and upper secondary education – a unique initiative both within and outside the Nordic context [18]. Further, the Finnish Pupil and Student Welfare Act (1287/2013) [19] encompasses goals focusing on: health and well-being promotion; problem prevention (including substance use as a risk behaviour) and securing early support when needed; promotion of the cooperation towards students’ homes; and multidisciplinary co-operation in the area of student welfare. This is realized in part through the student welfare services, which constitutes an intersectoral collaboration between schools, social and health care services, students and their parents/guardians, and also with other parties as needed – coordinated at the municipal level [19]. A distinct feature of this system is that the school welfare professionals are situated in the school arena (or ambulatory between schools), as opposed to being physically placed outside the school setting within e.g. municipal health services.

This study aims to explore the views of educational personnel and school welfare professionals regarding roles and responsibilities in substance use prevention in the Finnish school setting.

## Material and methods

### Design

The current study is based on a focus group data set collected within the scope of the EDGAR research project to support the development of a new model for substance use prevention in the Finnish Ostrobothnia region. Twenty-two baseline focus group interviews were conducted with school representatives prior to the introduction of the new model, aiming to capture the collective experiences regarding perceived challenges and supportive elements in substance use prevention. The current study is based on this focus group data, specifically exploring emerged views on responsibilities and roles in relation to primary prevention. In this regard, the study can be considered a secondary analysis to some extent [20]. Ethical approval for the study was obtained from the Board for Research Ethics at Åbo Akademi University on 11 November 2018. Written informed consent to participate in the research study was obtained from all participants.

### Participants and recruitment

The 22 focus groups were conducted separately for educational personnel and student welfare representatives during February–June 2019. The interviews took place at the schools. Participants were self-selected, recruited through the distribution of study information to nine basic education schools and four vocational or general upper secondary schools. Eleven focus groups were conducted with teaching personnel, and eleven focus groups with school welfare representatives. Participant characteristics can be seen in Table 1. All groups encompassed 3–4 participants, with the exception of two groups of school welfare personnel that differed in size (two and eight participants) due to last-minute cancellations or a keen interest in study participation. Two researchers functioned as moderators in each of the focus groups.

**Table 1.** Characteristics of the 74 focus group participants.

School welfare personnel		Educational personnel	
School nurses	11	Teachers	28
School counsellors	13	Special education teachers and assistants	6
School psychologists	4	Study advisors	3
Other	2	Principals	6
		Municipal level school authority representative	1
Number of years of experience in the field, mean (range)	11.0 (0.5–33.0)	Number of years of experience in the field, mean (range)	18.7 (1.0–34.0)
Female professionals	28 (93%)	Female professionals	29 (65.9%)

### Data collection and analysis

The interviews were semi-structured, utilizing an interview guide encompassing a broad set of questions regarding perceived challenges and possibilities in primary substance use prevention (see Appendix A for interview guide). The discussions, lasting between 31 and 70 min, were digitally recorded. The transcripts totalled 276 pages.

**Figure 1.**
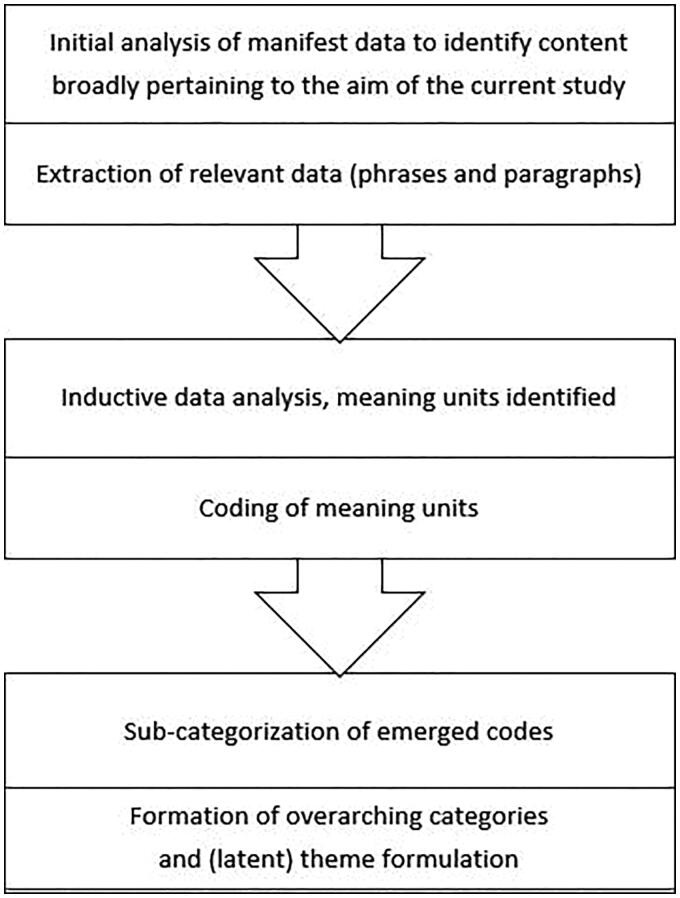
An outline of the qualitative content analysis process.

For the purposes of this study, the data set was systematically screened multiple times for phrases and paragraphs broadly pertaining to the focus of the current study (i.e. perceptions concerning roles and responsibilities). After this initial screening process, where relevant phrases and paragraphs were identified and extracted from all group discussions, qualitative content analysis was performed [21–22]. The identified and extracted content was condensated and coded in an inductive process where sub-categories and main categories were formed, based on manifest data (see Figure 1). The proposed categorizations were discussed and finalized in a dialogue between the authors. Both authors have experience in conducting focus group studies.

## Results

**Figure 2.**
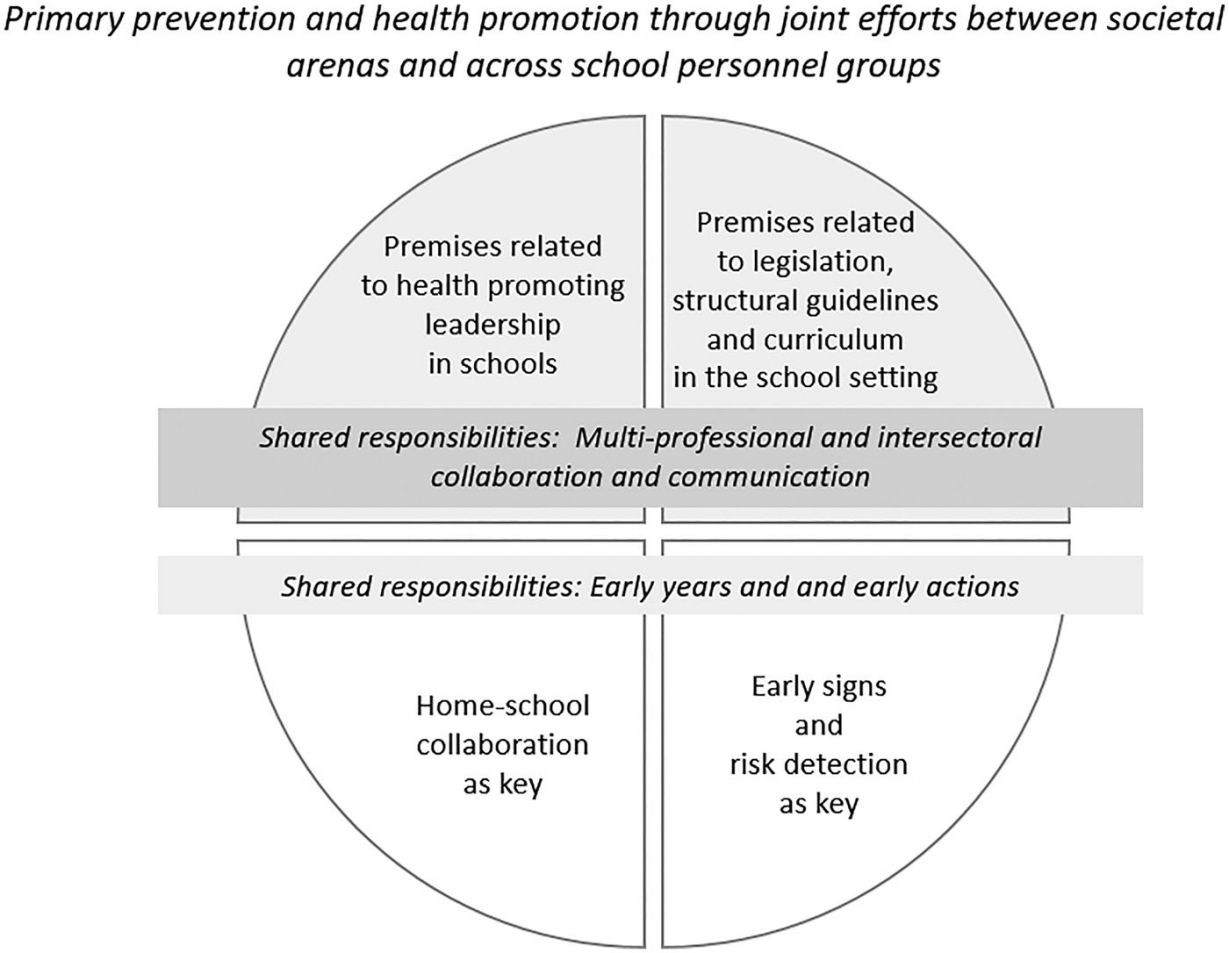
An overview of the emerged study results, encompassing the overarching theme, categories and sub-categories.

The analyses resulted in two categories, four sub-categories and an overarching theme (see Figure 2). While the focus group interviews focused on primary prevention of substance use, many participants focused their discussion on health promotion, especially mental health promotion. Various factors contributing to shared responsibility and continuous joint efforts in mental health promotion and risk prevention efforts emerged, together with issues perceived to complicate or impede cooperation and shared responsibility. The views of the participants at times reflected the age of the student group they were working with. However, many participants referred more broadly to the school setting in their reflections, not limiting discussions to a particular age group.

### Shared responsibilities: Multi-professional and intersectoral collaboration and communication

#### Premises related to health-promoting leadership in schools

The participants generally agreed that primary prevention of substance use and health promotion and support of the development and wellbeing of children and youth is a shared responsibility between arenas, on a broader municipal or regional level. However, several participants mentioned how municipal resources in the form of appointed persons focusing on primary prevention or health promotion felt less prioritized (e.g. such roles had disappeared in reorganizations). The participants described the role of various municipal, non-governmental or non-profit organizations and volunteer groups providing constructive, health-promoting activities for children and youth. The adults involved in these activities were considered as resources offering general support, functioning as (primarily positive) role models in relation to health and lifestyle. School representatives discussed the collaboration with these parties in health promotion and substance use prevention activities taking place within the school setting, targeting students, parents and/or personnel. While the study participants acknowledged the school setting as an important arena for health promotion and ill-health prevention, many school representatives also felt that an unreasonable responsibility and expectation was sometimes placed on the school, referring to the relevance of other contexts.

Considering issues within the school setting, some interview participants expressed perceived limitations in resources and a lack of prioritization of health promotion and primary prevention. This was related to a concern that activities they want to or should be doing, focused on health promotion directed at the whole student population, oftentimes felt non-prioritized. Here, the assigned roles and responsibilities were considered a challenge. For example, several school welfare representatives believed that they could contribute more to the universal health promotion and primary prevention work targeting the whole student body, but felt prohibited or limited to do so in accordance with their current work description.

I would like to have more time out in the classroom or leading groups. Now there is a lot of time dedicated to discussions with individual students […].

(School welfare personnel representative)

However, there was a variation here with some study participants expressing how the school leadership prioritizes health promotion and the creation of a supportive environment by allocating resources specifically for these purposes or promoting these aims in other ways. While substance use prevention was often framed as distinct informational activities focusing on the features of a specific substance (aimed at students and/or parents), several school representatives highlighted how they within the scope of their roles contributed to strengthening protective factors for health, which was regarded as equally important to prevention targeting risk factors for ill-health. In this way, primary prevention of substance use was seen as connected to universal (mental) health promotion contributing to students’ overall health and wellbeing, embedded in the overall workings of the school. This included creating a constructive and encouraging school atmosphere, emphasizing communication and dialogue with students - with some highlighting drug-free messages as a part of this -, promoting social inclusion and mutual respect and care between students in the framework of, e.g. anti-bullying work, seeing and hearing students, and focusing on building students’ self-esteem and mental health. Hence, substance use prevention was by many seen as part of a broader picture.

I see it as part of a bigger whole. I don’t think you can pick out substance use prevention as an isolated thing, it’s ingrained in everything else we do.

(Teaching personnel representative)

Similar to the perceived significance of inter-sectoral collaboration, the importance of multi-professional collaboration within the school context was emphasized. School representatives described various collaborative procedures and activities, where many stressed good cooperation and mutual support in their work and shared care for students. School personnel creating a health-promoting network around students, and the students having multiple adult resources as role models and confidants in the school were emphasized. However, some teacher representatives also highlighted challenges in relation to their roles and questioned how much involvement was appropriate. Also, some school welfare personnel felt that they would benefit from more communication with teachers, as the collaboration sometimes was felt to be limited to handling specific incidents (such as a student found smoking).

#### Premises related to legislation, structural guidelines and curriculum in the school setting

Structural guidelines related to substance use prevention and health promotion were viewed as affecting the day-to-day activities within the schools, also supporting common messages and rules. These ranged from legislation (e.g. school grounds as non-smoking areas) to recommendations and school policy concerning for example age limits for energy drink consumption. Also related to legislation, some informants described being part of legally required municipal fora and multi-professional working groups focusing on substance use prevention, while others did not participate in these or had no knowledge of them. Some found the networking and working groups to be helpful and important in supporting intersectoral efforts, while others participating in (or acquainted with) these fora felt that the purpose or benefits of these groups was unclear or lacking, articulating e.g. a need for the work to be more pragmatic. In some instances, previous working groups had disappeared due to reorganizations or shifts in leadership which disrupted important collaboration continuity. Regarding the related municipal plans guiding substance use prevention, some informants expressed a need for municipal plans - describing various activities, processes and related roles and responsibilities - to be developed as these did not exist, while others felt that updating the plans themselves took an unreasonable amount of time and questioned why each municipality needed their own specific plan.

Further, many school representatives referred to the Pupil and Student Welfare Act and the obligations regarding having a student welfare group in the school, responsible for the substance use plan and student welfare plan. The perceived adherence to these guidelines varied, with some stating that their school had not yet developed the plans or gathered the responsible working groups. Similar to municipal plans, some participants questioned why schools needed separate substance use prevention plans and the time and resources it took to update these. On the other hand, while a lack of structures was highlighted in some schools, in others the restrictions or limitations associated with the Pupil and Student Welfare Act and related regional plans and initiatives were emphasized, with participants wishing for more flexibility and independence in creating ideal structures and procedures.

[…] but then it’s immediately ”well if school welfare personnel in (specific school) are doing this then these schools also have to do this” and then it sort of becomes impossible because it grows so big and falters. No one has the energy to address it. There needs to be that opportunity where you say “I am working in this school and now I am seeing this problem, now I am going to deal with this here”. (School welfare personnel representative)

Among educational personnel especially, a lot of focus was placed on the curriculum. Some respondents highlighted how all teachers in the school setting should be able to discuss substance use-related themes with students, in line with a focus on broader horizontal themes in the curriculum. The views on how prepared or comfortable teachers in different subjects were to raise the theme varied, and time restraints and stress related to incorporating substance use-related themes were highlighted. Many welcomed the relatively new subject of health education, perceived as providing a natural, more continual forum for discussing substance use and related issues with students, in addition to broader health themes. This was regarded positively, as theme days or occasional lectures were seen as too limited in scope. However, several of the participants teaching health education did not feel confident or positive about holding classes related to substance use and felt an external person should be responsible for the theme.

Health education is difficult in the sense that it’s always me who is teaching that group, it’s me who is placing my values or the schools and subjects values on the students in a way. They take it to heart in a different manner if it’s sometimes someone external […].

(Teaching personnel representative)

Other limitations concerned health education being integrated with other subjects in the lower grades, limiting its focus. Here some said more focus should be placed specifically on, e.g. positive psychology and self-esteem in lower grades. In upper secondary school, on the other hand, not all health education courses are compulsory - hence interrupting continuity. The issue of who was to discuss the topic varied – some wished to have an external party, NGO representative, police representative or similar discussing the theme. This again reflects the focus placed by many on informative activities as constituting prevention.

### Shared responsibilities: early years and early actions

#### Home-school collaboration as a key

Participants in all categories considered the homes as an important arena, highlighting the importance of the family before children enter the school setting, and parents influencing children’s lifestyle and health directly and indirectly throughout life. Informants placed importance on the homes and caregivers’ health-promoting roles in caring for and supporting their children. This included the mention of parents’ responsibilities in establishing open and constructive parent-child communication, general parental involvement, and parents contributing to children’s development and self-esteem through e.g. supporting their engagement in constructive free-time activities. The participants mentioned parents as role models, and values and attitudes in the home as important in relation to lifestyle and health, including substance use. Some school representatives pinpointed the home as the most important arena and felt that more emphasis needed to be placed on parents.

I appreciate these projects, but they are always targeting us working in the schools. […] I miss the parents here, what are they supposed to be doing? Or what do we think parenting encompasses?

(School welfare personnel representative)

Participants from the different groups also stressed that parents could promote children’s health by communicating and networking amongst one another, and for example setting mutual boundaries and standards. Parents needing to stay informed about issues related to children’s health and wellbeing, including substance use-related issues were likewise raised. The need to support parents was also underlined – both in relation to general support and information but also support if challenges arose in a family, again, starting already from child welfare clinics.

The school was considered a key arena to reach parents through e.g. arranging events focusing on themes related to students’ health and wellbeing, including substance use, and providing a forum for communication and support between parents. Many school representatives received positive feedback from parents related to these initiatives. The school could also help parents to raise topics related to mental health or substance use with children of different ages or refer parents to useful information or support resources. While some school representatives engaged in these forms of activities, others were critical to these expectations, feeling that they either did not have the tools for this or that supporting the whole family was not possible in their current role.

It’s stated so nicely in our instructions that we should support the whole family and so on… Yes, but how am I supposed to do that? We don’t really have any means to do that. So that’s something that looks good on paper.

(School welfare personnel representative)

The need to focus on the early years and early actions to support health also encompassed the need for working on health and mental health promotion among children of lower ages in early childhood (pre-school) education and basic education, together with more specific substance use prevention approaches.

#### Early signs and risk detection as a key

It was perceived by many respondents that the educational personnel had the primary responsibility for noting and reacting to various warning signals indicating substance use, or more generally that students were unwell.

You can’t place all the responsibility on the school welfare personnel either, because there are others of us here also, important adults, who I think need to be observant.

(Teaching personnel representative)

This included school absence, notable changes in a student’s study performance and similar. Resource-related challenges were however raised here by some teaching personnel, referencing, e.g. a large number of students. Many school welfare representatives viewed teachers as attentive and reactive, while others wished for teachers to be more vigilant and communicative towards the school welfare personnel. Depending on the students’ age and related implications for consent, teachers were expected to initiate processes with the home and/or student welfare services, encompassing the compulsory gathering of expert groups, which in turn required student consent. A major challenge to the intra-school communication was the issue of the Pupil and Student Welfare Act and more specific regulations related to confidentiality and consent. While the rationale for these regulations was understood, general differences in the interpretation of rules posed a challenge, together with the fact that they were deemed to lessen communication within the school and towards the homes (doubts over who one could share an eventual concern for a student with). For example, the fact that 18-year olds have the right to prohibit the school from contacting their parents was not necessarily felt to be in the interest of the student. Some participants felt that the regulations contributed to some students ‘slipping between their fingers’ and not knowing what processes other personnel may have initiated. Student welfare personnel also perceived themselves as having responsibility for initiating processes, but with a greater focus on addressing concerns raised by educational personnel. These were to a greater extent secondary or tertiary efforts (often involving external parties e.g. social services) where the process was seen as more clear in contrast to primary prevention and health promotion activities and planning.

The importance of home-school collaboration also came up specifically from a risk perspective. It was perceived that school personnel and activities could serve to counteract eventual negative influences and risk factors in the home environment, as parental roles and responsibilities were not always met. Some school welfare representatives felt that problems in parental roles not being fulfilled have increased in recent years, with the mention of parents treating youth more like friends and avoidance of conflicts with their children. More specifically related to substance use prevention, many teachers expressed frustration over parents condoning substance use among under-aged students and not supporting substance use prevention in the school, challenging school messages and communication.

For many of those who are caught smoking, the parents say ”of course he can smoke”. What does the school do then? Then that’s how it is. Parents are the most important, that’s just how it is. They create the foundation and we can’t change basic values in the school.

(Teaching personnel representative)

Further, many parents (especially many of those who school representatives felt should be present) did not participate in events and activities arranged by the school. This was viewed as being related to larger issues of societal polarization where many students experiencing ill-health were perceived to come from families with more extensive social challenges and struggles in providing a constructive and supportive environment for children. The issue of a smaller, but growing, group of marginalized young people exhibiting more extensive mental health problems was also raised – and especially the challenge in responsibility related to supporting these students, who previously were identified in the school context but now are not reached due to school absence and non-response to outreach efforts.

## Discussion

While the focus group study focused on primary prevention of substance use (minimizing and mitigating risk factors), a lot of participants focused on health promotion (centering around strengthening resources) – and primarily mental health promotion - in their discussions. Hence, the results showcase participants’ experiences of responsibility and roles related to both primary prevention of substance use and mental health promotion in the school setting.

The expressed emphasis on joint effort reflects earlier propositions concerning the importance of creating supportive, health-promoting environments at all levels and how intersectoral action and collaboration with outside agencies are central to effectiveness in mental health promotion and problem prevention interventions in schools [23–24]. However, respondents experienced that less resources are placed on health promotion and primary prevention in municipalities and schools, with secondary prevention prioritized due to limited resources. Role descriptions and responsibilities concerning secondary prevention (protocols, processes) were also regarded as more clear. School leadership in health promotion and substance use prevention could be strengthened by a stronger focus on practical legislation implementation in this area. This is interesting to consider in relation to the Pupil and Student Welfare Act, placing a broad focus on health promotion. Earlier evaluation of the act, performed in 2017 [25], shows that the first aim related to the promotion of student health, social well-being and studying capacity has not shown much progress in practice. With regard to the second aim related to primary prevention and early support, the development of the preventive approach has likewise been lagging in basic education and general upper secondary schools. For the vocational school setting, the limited impact was likewise noted, but health promotion (for example concerning mental health) and problem prevention was at a better level [26]. The results of the current study point to persisting challenges in the translation of the aims of the act into practice. Regarding specific aspects of the Pupil and Student Welfare Act (e.g. multi-professional student welfare groups and related plans), confidentiality and consent regulations were identified as major challenges to intra-school communication and collaboration and cooperation with the homes. A similar finding emerged in a Norwegian study [27], where teachers – although considering themselves front-line gatekeepers in supporting students’ mental health and identifying mental health problems – felt that inter-professional collaboration was challenged by confidentiality restraints.

Health education being included in the curriculum was perceived as positive in relation to primary prevention and health promotion efforts, in line with a whole-school approach [25], albeit it was suggested that health education could be further emphasised in the first years of basic education. Interestingly however some of those teaching health education felt that it was beneficial to outsource the specific theme of substance use to someone external. This finding in part reflected that while primary prevention of substance use to a large extent was described in terms of mental health promotion, it was also at times framed as being limited to informative activities focusing on specific substances (e.g. specific psychoactive properties). This result reflects the findings of an earlier systematic review of factors affecting the implementation of substance use interventions in secondary schools [28]. In the review studies (none of which took place in the Nordic countries), an emerged challenge was the issue of educational personnel feeling uncomfortable with the substance use theme - referring to a need for external expertise and the view that substance use prevention falls somewhat outside of their perceived role. In the countries of the review studies, however, health education is not a stand-alone subject as opposed to the situation in the Finnish setting.

While the original study focus was primarily on the school setting, the importance of early health promotion and primary prevention initiatives starting at earlier ages and the importance of the homes was highlighted extensively. This is in line with the findings of an earlier Finnish study showing how parents and educational personnel assigned a slightly larger role to parents (compared to teachers) in informing children on substance use [16]. The results also reflect earlier Finnish research evidence concerning views on health promotion and education as a shared responsibility between schools and homes, with greater emphasis placed on parents [29].

Liaison with parents and parenting education have earlier been identified as characteristics of effective mental health promotion and problem prevention interventions in schools [24]. School representatives generally found parents or legal guardians to be actively engaged and cooperative and concerned if eventual problems arose. The homes could however also challenge school health promotion and primary prevention efforts through parents not functioning as role models, not supporting school health promotion and prevention efforts aimed at the whole student body, not participating in school-based events aimed at parents and not supporting school interventions when issues emerge. Related to this, broader issues of increasing social polarization were raised, along with challenges concerning marginalization among youth – issues perceived to be increasing also in this Nordic, egalitarian context. This was somewhat reflected in the educational institutions, i.e. school personnel expressing a perceived difference in the challenges facing students (and their families) in vocational school versus students in general upper secondary school.

A mutual understanding of the importance of early actions in the form of universal mental health promotion and primary prevention initiatives was found in this study. At the same time, the informants’ views and experiences show that the responsibility issue is complex, covering both unclarity of the primary prevention and health promotion concepts and what these entail, as well as varying perspectives on priorities needed in the mental health work within the school setting.

### Strengths and limitations

The 74 study participants constituted a relatively heterogeneous group in terms of their roles in the schools, thus contributing with different perspectives on substance use prevention in the school setting. While participants were self-selected, the sampling was somewhat purposeful in order to include professionals working with children and adolescents of different ages in schools of varying sizes in the study region.

In order to capture the collective experiences of school representatives, a focus group method encompassing small groups was deemed appropriate. While views on responsibilities and roles were featured in the group discussions, this was not the main theme of the interviews as such, which could potentially have affected results and constitute a source of bias. Because the issue of roles and responsibilities was a theme that was highlighted in all discussions the current study focus was however considered to be justified.

## Conclusion

While the school setting was viewed as an important arena for health promotion and primary prevention work targeting substance use and mental wellbeing, the importance of coordinated intersectoral efforts and especially the collaboration with the homes was emphasized. This in part due to the perceived need for early actions before children even enter the school setting. However, optimal intersectoral and intra-school collaboration warrants a common understanding regarding the roles and shared responsibility within and across personnel and stakeholder groups. This also entails a shift of focus from informative activities as the primary goal of primary prevention work – to a holistic approach where achieving mental health and preventing related potential risk behaviours related to substance use among young people are viewed as a continuous process, for which all adults in the young people’s lives are mutually responsible. Further research is needed on how to best support home-school and intersectoral collaboration in the mental health promotion and primary prevention work targeting young people and their families. This also encompasses the question of how to best support the implementation of current legislation.

## Appendix A

Sample interview questions utilized in the semi-structured interviews, focusing on experiences concerning substance use prevention in the school setting.

*Introductory question*

- What is your general view on substance use prevention in schools?

*Possibilities* (sample questions)

- Are there aspects of substance use prevention that work well?
- What factors do you feel support substance use prevention in your own work?

*Challenges* (sample questions)

- Are there any particular challenges affecting substance use prevention from your point of view?
- Is there anything you feel is difficult with regard to substance use prevention?
- Are there any factors you find particularly challenging? Could anything be done to avoid or address these challenges in your view?

*Development needs and possibilities* (sample questions)

- Is there anything you feel could be developed with regard to substance use prevention work in schools in general? Or in relation to your own job?
- Do you wish for any form of support in your work, in relation to substance use prevention?

## References

[1] ESPAD Group. ESPAD Report 2019: Results from the European School Survey Project on Alcohol and Other Drugs. Luxembourg: EMCDDA Joint Publications, Publications Office of the European Union; 2020.

[2] United Nations Office on Drugs and Crime (UNODC) and World Health Organization (WHO). International standards on drug use prevention. 2nd ed. Vienna: UNODC; 2018.

[3] MacArthur G, Caldwell DM, Redmore J, et al. Individual-, family-, and school-level interventions targeting multiple risk behaviours in young people. Cochrane Database Syst Rev. 2018;10(10):CD009927. 30288738 10.1002/14651858.CD009927.pub2 PMC6517301

[4] Tancred T, Melendez-Torres GJ, Paparini S, et al. Interventions integrating health and academic education in schools to prevent substance misuse and violence: a systematic review. Syst Rev. 2018;7(1):227. 30522529 10.1186/s13643-018-0886-3 PMC6284294

[5] Ballester L, Amer J, Sánchez-Prieto L, et al. Universal family drug prevention programs. A systematic review. J Evid Base Soc Work. 2021.;18(2):192–213. 10.1080/26408066.2020.1822976 32985382

[6] Biglan A, Van Ryzin MJ. Behavioral science and the prevention of adolescent substance abuse. Perspect Behav Sci. 2019;42(3):547–563. 31976449 10.1007/s40614-019-00217-y PMC6769129

[7] Tremblay M, Baydala L, Khan M, et al. Primary substance use prevention programs for children and youth: a systematic review. Pediatrics. 2020;146(3):e20192747. 32769198 10.1542/peds.2019-2747

[8] Haines-Saah RJ, Mitchell S, Slemon A, et al. Parents are the best prevention? Troubling assumptions in cannabis policy and prevention discourses in the context of legalization in Canada. Int J Drug Policy. 2019;68:132–138. 30025898 10.1016/j.drugpo.2018.06.008

[9] Walter KO, Paulo JR, Polacek GN. Faculty perceptions of their roles in alcohol education/prevention. J Drug Educ. 2013;43(2):173–182. 25068169 10.2190/DE.43.2.e

[10] Wyman J, Price JH, Jordan TR, et al. Parents’ perceptions of the role of schools in tobacco use prevention and cessation for youth. J Community Health. 2006;31(3):225–248. 16830508 10.1007/s10900-005-9010-4

[11] Small SP, Kushner KE, Neufeld A. Smoking prevention among youth: a multipronged approach involving parents, schools, and society. Can J Nurs Res. 2013;45(3):116–135. 24236375 10.1177/084456211304500308

[12] Gates PJ, Norberg MM, Dillon P, et al. Perceived role legitimacy and role importance of Australian school staff in addressing student cannabis use. J Drug Educ. 2013;43(1):65–79. 24855884 10.2190/DE.43.1.e

[13] Harris GE, Jeffery G. School counsellors’ perceptions on working with student high-risk behaviour. Can J Couns Psychother. 2010;44(2):150–190.

[14] Van Hout MC, Foley M, McCormack A, et al. Teachers’ perspectives on their role in school-based alcohol and cannabis prevention. Int J Health Promot Educ. 2012;50(6):328–341.

[15] van der Sar R, Brouwers E, van de Goor I, et al. Comparison between Dutch and Norwegian parents regarding their perceptions on parental measures to prevent substance use among adolescents. Addict Res Theory. 2014;22(1):68–77.

[16] Sormunen M, Goranskaya S, Kirilina V, et al. Home and school responsibilities for children’s health literacy development: the views of Finnish and Russian parents and teachers. Russ J Commun. 2018;10(1):70–90.

[17] Volmari K. Basic education in the Nordic Region. Similar values, different policies. Helsinki: Finnish National Agency for Education; 2019.

[18] Välimaa R, Kannas L, Lahtinen E, et al.Finland: innovative health education curriculum and other investments for promoting mental health and social cohesion among children and young people. In: Social cohesion for mental well-being among adolescents. Copenhagen: WHO Regional Office for Europe; 2008.

[19] Pupil and Student Welfare act (30.12.2013/1287) [cited 2020 Nov 20]. Available from: https://www.finlex.fi/sv/laki/ajantasa/2013/20131287#a14.12.2017-886 (Swedish).

[20] Heaton J. Secondary analysis of qualitative data: an overview. Hist Soc Res. 2008;33(3):33–45.

[21] Krippendorff K. Content analysis: an introduction to its methodology. 4th ed. Thousand Oaks (CA); Sage publications: 2018.

[22] Graneheim UH, Lundman B. Qualitative content analysis in nursing research: concepts, procedures and measures to achieve trustworthiness. Nurse Educ Today. 2004;24(2):105–112. 14769454 10.1016/j.nedt.2003.10.001

[23] Jackson SF, Perkins F, Khandor E, et al. Integrated health promotion strategies: a contribution to tackling current and future health challenges. Health Promot Int. 2006;21(suppl_1):75–83. 17307960 10.1093/heapro/dal054

[24] Weare K, Nind M. Mental health promotion and problem prevention in schools: what does the evidence say? Health Promot Int. 2011;26(Suppl 1):i29–i69. 22079935 10.1093/heapro/dar075

[25] Summanen A-M, Rumpu N, Huhtanen M. Evaluation of the implementation of the pupil and student welfare act in pre-primary education, basic education and general upper secondary education. Tampere; Finnish Education Evaluation Centre (FINEEC): 2018.

[26] Frisk T, Hietala R, Kies J. Evaluation of the implementation of the Pupil and Student Welfare Act in vocational education and training. Tampere; Finnish Education Evaluation Centre (FINEEC): 2018.

[27] Ekornes S. Teacher perspectives on their role and the challenges of inter-professional collaboration in mental health promotion. School Ment Health. 2015;7(3):193–211.

[28] Waller G, Finch T, Giles EL, et al. Exploring the factors affecting the implementation of tobacco and substance use interventions within a secondary school setting: a systematic review. Implement Sci. 2017;12(1):1–18. 29137649 10.1186/s13012-017-0659-8 PMC5684739

[29] Sormunen M, Tossavainen K, Turunen H. Parental perceptions of the roles of home and school in health education for elementary school children in Finland. Health Promot Int. 2013;28(2):244–256. 22373543 10.1093/heapro/das004

